# The Possibility of Vaccination against the Exanthemata

**Published:** 1915-12

**Authors:** 


### BUFFALO MEDICAL JOURNAL

**A Monthly Review of Medicine and Surgery**

EDITOR AND PUBLISHER, DR. A. L. BENEDICT, 228 Summer St., corner of Elmwood Ave., Buffalo, (Address for all communications. Please make personal and telephone calls before 1 P. M.)

Third, in age, on the western continent. Independent, but supports professional organization. News limited mainly to Western N. Y. and Penn.

Subscription $2.00 a year; $3.00 for two years in advance. Changes and errors in address or failure or duplication of delivery, should be reported immediately.

Yearly Volume 71. DECEMBER, 1915. No. 5

### The Possibility of Vaccination Against the Exanthemata.

Very early in our professional career, when we were deeply impressed with the scientific side of medicine, a side not long developed and more startling in contrast with the older form of empiricism, still common at that time, than at present when it is better developed but less conspicuous, a retired physician tried to impress upon us that the great problem before the medical profession was the control of scarlet fever. We remember, with shame, the scorn which we tried to conceal out of deference to his years. With longer experience, in spite of devotion to an entirely different line of professional interest, we are inclined to think that the opinion was and is correct. To a large degree, other problems which, at the time mentioned, seemed far more important, have been solved Many infections have been practically exterminated. The prophylaxis of others, as typhoid, tuberculosis, the venereal diseases, is quite clearly understood, only inertia and lack of practical means preventing their full applications. In regard to many diseases, of the same or different nature, we seem to lack only the development of technic or success is prevented by degenerative changes, largely inevitable, or else the problem, while just as important and just as far from solution for the actual case, involves a relatively insignificant proportion of the race.

Barring accidental escape, the exanthemata, excepting variola, still attack nearly all members of any large community. Having occurred, they confer a nearly absolute, life-long immunity, justifying the term semelincident, too little used. For these reasons, by the law of chance, they are preeminently children's diseases, as was variola (accented on the **i**, if you please) before its artificial and nearly perfect prophylaxis. While all the exanthemata deserve serious consideration, scarlet fever and variola are the only ones having a high mortality —approximately 10%. Excepting that we have generally succeeded in enforcing better and prompter care of patients, have appreciated better the necessity of guarding against sequelae, and have discarded certain older methods that were more or less harmful, it can not be claimed that we have made any progress in the thereapeutics of these diseases. Even smallpox, against which we have almost perfect prophylaxis, is not more successfully **treated** now than many years ago, perhaps less so, from relative lack of experience.

It is important to realize that **vaccination** in the limited sense, is an entirely different process from vaccination against bacterial diseases in general, or from the use of any kind of serum. Vaccinia has been proved to be a mitigated variola. Whether or not, the beautifully complicated life cycle of the more or less hypothetic variola protozoan, exists in fact or in fancy, the fact remains that antivariolar vaccination sets up a mitigated dwarf disease, very rarely serious and with a mortality in millionths or less, instead of percentages. Yet this dwarf disease confers an immunity equal for several years to that of typic variola and, with rare exceptions, sufficient to insure that even with neglect of repetition, infection will result only in atypic mild varioloid.

Now it is obvious that vaccination against other diseases, in the sense pertaining to variola, is limited to semelincident diseases and cannot apply to diphtheria, gonorrhoea, malaria, tuberculosis, or any other disease which does not protect against its own recurrence.

Furthermore, if the life-cycle theories are correct, there can be no true vaccination against a bacterial disease, even if practically semelincident like typhoid.

It is, therefore, among the exanthemata—and possibly some protozoan diseases comparatively rare in this country—that we must look for any close analogue of antivariolar vaccination and, as stated, it is only in regard to scarlet fever that the danger assumes proportions which render the problem of transcendent humanitarian importance, though its ultimate applicability to measles, must also be considered.

It has already been claimed, though with even less evidence than for the variolar organism, that the other exanthemata are due to analogous protozoa, which pass through analogous life cycles.

Smallpox produces mitigated types in bovines, equines (grease), and ovines. Whether horse and sheep pox may be used practically in place of vaccinia, is a question which has, apparently, not been decided but which is decidedly academic in view of the very satisfactory results of bovine virus.

Anyone who will carefully go over a check list of infections will be convinced that scarlet fever and the remaining exanthemata are the only ones in which the two requisites of semelincidence and possibility of modification of life cycle of the germ, in connection with the practical consideration of wide incidence among civilized people, are fulfilled. We need not quibble as to whether it is proper to term the introduction of killed or scotched germs **vaccination** or not, and we may be very hopeful as to the temporary or even prolonged prophylactic value of germ products of all kinds, when better technic has been attained. But for any true analogue of antivariolar vaccination, we are limited to the exanthemata (or to diseases of little interest to us in this country) and, for practical, numeric reasons, to scarlet fever.

Reduced to bare practical considerations, the problem is to find an animal susceptible to scarlet fever, but in mitigated form, whose virus, again inoculated into man, will reproduce the disease in practically the same dwarf form.

While we would not venture to predict that such a disease exists, there are some reasons in its favor. Few or no infections are limited to a host of a single species, though certain diseases, as typhoid and gonorrhea, are practically restricted to man, so far as spontaneous origin is concerned. Natural immunity to scarlet fever, in the face of marked exposure, is quite freely observed. Such immunity, for semelincident diseases generally, is not hereditary in the true sense, though it may occur from maternal infection during pregnancy. But, in the great majority of such infections, abortion occurs and, if it did not, a history to account for the immunity of the child, would almost always be obtainable. While, of course, resistance to exposure does not necessarily imply any true immunization, it is strongly suggestive. Again, scarlet fever quite often develops when it cannot be traced to a human source. This argument is obviously weak ,still it suggests an accidental protection analogous to cowpox.

Assuming that scarlet fever does occur in a lower animal, in some type, we should, on general principles, expect that the animal would be a common one, though not necessarily closely related to man zoologically. Certain observations as to the period of inoculability of yellow fever, for instance, suggest that the stegomyia is not an accidental carrier but that it actually has to develop the same disease, itself. Again, if there is an occurrence of scarlet fever in a lower animal—using the word animal in a broad sense—it is probably in a mitigated form. The association of children with domestic animals is so general that the occurrence of a fatal or even conspicuous disease in the latter could scarcely have been overlooked. Even if the host were any common insect or vermin, the occurrence of a serious epizootic in the wake of scarlet fever in the human being, would be conspicuous by the deaths.

